# Administration Routes and Doses of the Attenuated African Swine Fever Virus Strain PSA-1NH Influence Cross-Protection of Pigs against Heterologous Challenge

**DOI:** 10.3390/ani14091277

**Published:** 2024-04-24

**Authors:** Mikhail Vlasov, Irina Sindryakova, Dmitriy Kudryashov, Sergey Morgunov, Olga Kolbasova, Valentina Lyska, Sergey Zhivoderov, Elena Pivova, Vladimir Balyshev, Sanzhi Namsrayn, Timofey Sevskikh, Alexey Sereda, Denis Kolbasov

**Affiliations:** Federal Research Center for Virology and Microbiology (FRCVM), Academician Bakoulov Street, Bldg. 1, 601125 Volginsky, Russia; vlasovmikhail1993@yandex.ru (M.V.); dima_kudryashov@mail.ru (D.K.); dohliy55555@mail.ru (S.M.); olgakolbasova@gmail.com (O.K.); vliska@yandex.ru (V.L.); zhivoderov-serg@mail.ru (S.Z.); lenamail09@inbox.ru (E.P.); balyshevvm@rambler.ru (V.B.); namsrayn.szh@gmail.com (S.N.); sefskih@mail.ru (T.S.); kolbasovdenis@gmail.com (D.K.)

**Keywords:** African swine fever, non-hemadsorbing strain, heterologous protection

## Abstract

**Simple Summary:**

African swine fever (ASF) is a lethal hemorrhagic disease of *Suidae,* i.e., domestic pigs and wild boars, caused by African swine fever virus (ASFV). The development of cross-protective vaccines against ASF is imperative for effective disease control, particularly in regions where ASF is endemic, potentially featuring multiple circulating ASFV isolates. It was shown that the intranasal administration of a low dose of ASFV-PSA-1NH (immunotype IV, genotype I) to pigs led to minimal manifestation of clinical signs of ASF and the formation of a high level of protection against the heterologous strain ASFV-Stavropol 01/08 (seroimmunotype VIII, genotype II).

**Abstract:**

African swine fever (ASF) is a lethal hemorrhagic disease of *Suidae*, i.e., domestic pigs and wild boars, caused by African swine fever virus (ASFV). The development of cross-protective vaccines against ASF is imperative for effective disease control, particularly in regions where ASF is endemic, potentially featuring multiple circulating ASFV isolates. The investigation of non-hemadsorbing naturally attenuated isolates and laboratory recombinant strains with a deletion in the *EP402R* gene has attracted interest. Our study aimed to assess the impacts of various administration routes and doses of the naturally attenuated ASFV-PSA-1NH (immunotype IV, genotype I) isolate on the manifestation of clinical signs of ASF and the level of protection against the heterologous ASFV-Stavropol 01/08 strain (seroimmunotype VIII, genotype II). The results demonstrated that the intranasal administration of a low dose of ASFV-PSA-1NH to pigs minimized the clinical signs of ASF and established a high level of protection against the heterologous strain ASFV-Stavropol 01/08. Despite the challenges in standardizing the dosage for intranasal administration, this approach appears as a viable alternative in ASF vaccination.

## 1. Introduction

African swine fever (ASF) is a lethal hemorrhagic disease of *Suidae*, i.e., domestic pigs and wild boars. This can result in very high mortality and has a severe socio-economic impact on affected countries.

The African swine fever virus (ASFV) is a large, enveloped, and double-stranded DNA virus [1] with a genome size ranging between 170 and 195 kb [2,3,4]. It is the only member of the family *Asfarviridae* and the genus *Asfivirus* [5]. However, it shares certain characteristics with other members of the nucleocytoplasmic large DNA virus family, suggesting an evolutionary relationship [6]. ASFV expresses variability in virulence among domestic pigs, ranging clinically from highly lethal to subclinical manifestations [5,7].

The genome encodes between 150 and 167 open reading frames, depending on the virus strains [8]. To date, 24 ASFV genotypes have been reported worldwide based on the *B646L* gene, which encodes the capsid protein p72, all known to circulate in Africa [9,10,11]. Although ASFV genotyping is useful for some purposes, it does not fully correlate with the available data on cross-protection and may have limited value for predicting the effectiveness of vaccine cross-protection [12,13]. Based on the results of studying the antigenic and protective properties of isolates/strains, a seroimmunotype classification of ASFV was developed [14,15,16]. The isolates/strains of ASFV are divided into nine (I–IX) seroimmunotypes and three other small groups [15]. In 2015, a new approach was proposed, making it possible to predict with a high degree of probability the seroimmunotype/immunotype belonging to both hemadsorbing and non-hemadsorbing isolates of ASFV. This approach is based on sequencing and phylogenetic analysis of the *EP402R* gene [17].

Obtaining cross-protective vaccines is desirable for effective ASF control, especially considering areas where disease is enzootic and more than one ASFV seroimmunotype could be circulating. The best example of this complex epidemiological picture comes from southeastern Africa and the Iberian Peninsula from 1960 to 1994 [18,19,20,21,22].

In the context of creating heterologous live vaccines against ASF, significant interest lies in studying the protective properties of non-hemadsorbing naturally attenuated isolates and laboratory recombinant strains with a deletion in the *EP402R* gene [23,24,25]. King K. et al. (2011) demonstrated the formation of protection against heterologous ASFV isolates [12]. Immunizing pigs with the naturally attenuated non-hemadsorbing isolate of genotype I from Portugal, OURT88/3, provided protection against death upon subsequent infection with a closely related hemadsorbing virulent isolate, OURT88/1. Additionally, European domestic pigs immunized with OURT88/3 developed protective immunity against two virulent ASFV isolates—Benin 97/1 of genotype I and Uganda 1965 of genotype X. The naturally attenuated non-hemadsorbing genotype I isolate NH/P68, produced in porcine alveolar macrophages (PAMs), provided 100% protection against homologous (Lisbon60) and heterologous (Armenia07) challenges but with side effects in vaccinated animals, such as slightly raised body temperatures, necrotic skin areas, and joint swelling, associated with chronic ASFV infection [24].

Interestingly, the routes of administration and the viral infectious dose significantly influenced the immunization outcome with OURT88/3 isolate. Intranasal administration led to two clinical groups: pigs with periodic clinical manifestations of ASF (doses of 10^3^ and 10^4^ TCID_50_, 100% protection against homologous OURT88/1 isolate) and animals with a chronic form of ASF (dose of 10^5^ TCID_50_, 66% protection). Intramuscular immunization of pigs with low and medium doses (10^3^ and 10^4^ TCID_50_) resulted in a lower percentage of protection (50 and 66%) [26]. Studies with the naturally attenuated NH/P68 isolate showed that intranasal immunization induced a higher degree of protection than intramuscular administration [27].

In our study, we aimed to determine the impacts of different routes and doses of administration of the naturally attenuated isolate ASFV-PSA-1NH on the manifestation of clinical signs of ASF and the level of protection against heterologous ASFV-Stavropol 01/08. We showed that the intranasal administration of a low dose of ASFV-PSA-1NH (immunotype IV, genotype I) to pigs led to minimal manifestation of clinical signs of ASF and the development of a high level of protection against heterologous ASFV-Stavropol 01/08 (seroimmunotype VIII, genotype II).

## 2. Materials and Methods

### 2.1. Viruses

The ASFV strains and isolates were received from the collection of microorganisms of FRCVIM. The non-hemadsorbing ASFV isolate PSA-1NH (Portuguese Swine Attenuated-1 Non-Hemadsorbing, ASFV-PSA-1NH, immunotype IV, genotype I) was kindly provided to the organization by researchers from Portugal in 1978. It was likely a variant of the ASFV-NHAI isolate [28]. The virulent hemadsorbing ASFV strain Stavropol 01/08 (ASFV-Stavropol 01/08, seroimmunotype VIII, I genotype II) was isolated in Russia [29].

### 2.2. Animal Experiments and Ethics Statement

Two- to three-month-old female and male (20–30 kg) pigs of the Large White pig breed purchased from the Experimental Animal Preparation Sector of the FRCVM were used in the experiment. The experiments involving animals and viruses were performed in accordance with the National Institutes of Health’s Guide for the Care and Use of Laboratory Animals [30] and were approved by the Bioethics Commission of FRCVM. All experiments were performed in the Biosafety Level 3 facilities of the FRCVM and were conducted under the supervision of the institutional animal care and use and institutional biosafety committees. Animal care and procedures were performed in accordance with the guidelines of the Good Experimental Practices (GEPs) and under the supervision of the Bioethics Commission of the FRCVM [30].

### 2.3. Experimental Designs

In experiment 1, groups 1 and 2 consisted of five pigs each (animals 1–5 and animals 6–10). Group 3 (intact) consisted of three pigs (animals 11–13). On day 0, the pigs in group 1 were intramuscularly inoculated with ASFV-PSA-1NH at a dose of 10^5^ TCID_50_, while the pigs in group 2 were inoculated with a dose of 10^3^ TCID_50_, and those in the control group 3 were intramuscularly inoculated with PBS (phosphate-buffered saline). On day 28, all animals in groups 1–3 were intramuscularly infected with ASFV-Stavropol 01/08 at a dose of 10^3^ HAD_50_ (Supplementary Figure S1).

In experiment 2, groups 4 and 5 consisted of five pigs each (animals 21–25 and animals 26–30). Group 6 (intact) consisted of three pigs (animals 31–33). On day 0, the pigs in group 4 were intranasally administered ASFV-PSA-1NH at a dose of 10^5^ TCID_50_, while the pigs in group 5 were administered at a dose of 10^3^ TCID_50,_ and those in the control group 6 were intranasally administered with PBS. On day 28, all animals in groups 4–6 were intramuscularly infected with ASFV-Stavropol 01/08 at a dose of 10^3^ HAD_50_ (Supplementary Figure S2).

### 2.4. Clinical Evaluation

The severity of the disease was assessed by a quantitative clinical score (CS) obtained by adding the values for the clinical signs recorded on a daily basis [12]. Pre-determined humane endpoints included a pig displaying severe signs of fever, anorexia, recumbence, respiratory distress, and digestive signs for more than two consecutive days. A CS ≥ 6, based on the sum of minimum scores for signs 1–7, was considered as the borderline. Signs 8–10 were characteristic of the subacute and acute forms of ASF [12].

### 2.5. Sample Collection

Blood specimens from the anterior vena cava were sampled at 0, 3, 7, 10, 14, 21, 28, 31, 33, 35, 38, 42, 49, 56, and 63 days post-inoculation (dpi) with ASFV-PSA-1NH.

To obtain whole blood, the blood samples were collected in EDTA tubes (TD VIK LLC, Lyubertsy, Russia), and to obtain blood serum, the whole blood was collected in serum tubes (TD VIK LLC, Lyubertsy, Russia), allowed to clot, and then centrifuged for 10 min at 2000× *g*.

Three aliquots of all the samples described above were prepared and stored in 5 mL or 2 mL cryovials at minus 40 °C until testing (Corning Inc., Corning, NY, USA). All equipment used for sampling was cleaned and disinfected between pigs and uses. All samples were frozen and thawed once prior to the test.

Preparation of peripheral blood leukocytes cell (PBLC) culture was performed as described previously [16].

### 2.6. Determination of the Infectious Activities of ASFV

The infectious activities of the isolate/strain were determined by titration in three-day PBLC (four wells for each tenfold dilution). The results were examined by the presence of a cytopathic effect (TCID_50_) or hemadsorption phenomenon (HAD_50_) after 5–7 days.

### 2.7. Detection of Anti-ASFV Antibodies

Serum samples (0, 10,14, 21, 28, 35, 42, 49, 56, and 63 dpi) were tested in duplicates using the INgezim PPA Compac solid-phase ELISA test kit (INMUNOLOGÍA Y GENETICA APLICADA, S.A., Ingenasa, Madrid, Spain). According to the kit instructions, the status of each tested serum was expressed using the coefficient of inhibition (x%).

### 2.8. Real-Time Polymerase Chain Reaction (Real-Time PCR)

The viral DNA was extracted from all EDTA blood samples using the QIAmp_DNA Mini kit (QIAGEN, Hilden, Germany) according to the manufacturer’s instructions. The detection of ASFV genomic DNA was carried out according to the protocol described by Fernandez-Pinero et al. (2013) on Bio-Rad CFX 96 Real-Time Detection Systems (Bio-Rad, Hercules, CA, USA) [31]. Samples with Ct (cycle threshold) < 40 were considered as positive, while samples with Ct ≥ 40 and no Ct value were considered as negative.

### 2.9. Statistical Analysis

The statistical analysis of the results was performed using the GraphPad Prism software version 8.0.1. Differences between counts were considered significant at *p* < 0.05. The virus titers were calculated according to the method described by B.A. Kerber in I.P. Ashmarin’s modification [32].

## 3. Results

### 3.1. Immunobiological Indicators in Pigs after Intramuscular Inoculation of Different Doses of ASFV-PSA-1NH and Challenge with ASFV-Stavropol 01/08

**Figure 1:**
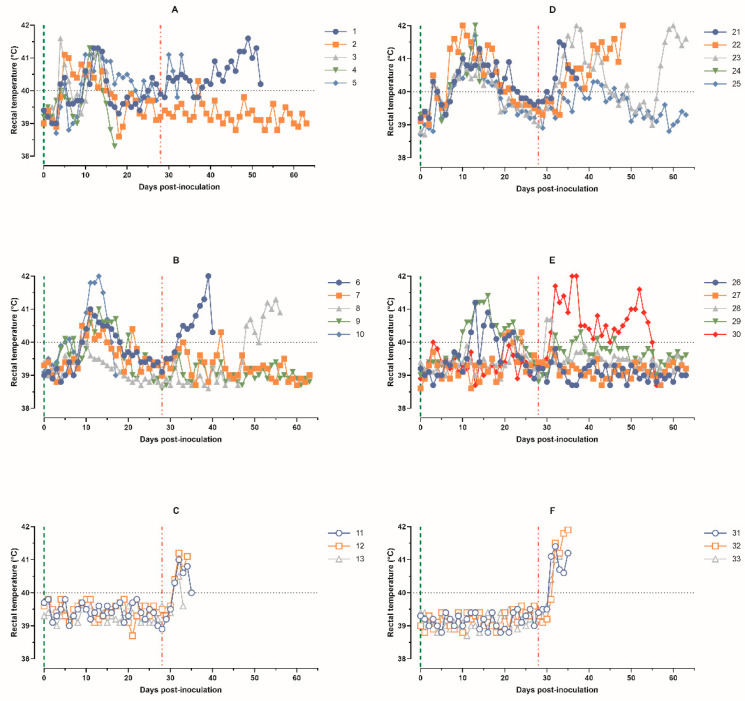
Kinetics of pig’s body temperature: (**A**) animals 1–5, group 1 (intramuscularly inoculated on day 0 with ASFV-PSA-1NH at a dose of 10^5^ TCID_50_); (**B**) animals 6–10, group 2 (intramuscularly inoculated on day 0 with the ASFV-PSA-1NH at a dose of 10^3^ TCID_50_); (**C**) animals 11–13, group 3 (control group intramuscularly inoculated on day 0 with PBS); (**D**) animals 21–25, group 4 (intranasally administered on day 0 with ASFV-PSA-1NH at a dose of 10^5^ TCID_50_); (**E**) animals 26–30, group 5 (intranasally administered on day 0 with ASFV-PSA-1NH at a dose of 10^3^ TCID_50_); and (**F**) animals 31–33, group 6 (control group intranasally administered on day 0 with PBS). All pigs were challenged at 28 dpi with ASFV-Stavropol 01/08. Each curve represents an individual animal’s values. Vertical dashed lines: green—days of inoculation/administration of ASFV-PSA-1NH;red—challenge with ASFV-Stavropol 01/08.

The intramuscular inoculation of ASFV-PSA-1NH at a dose of 10^5^ TCID_50_ to pigs in group 1 (animals 1–5) caused fever (body temperature ≥ 40.0 °C) in all animals from 4 to 27 dpi, lasting from 6 to 17 days. In four out of five pigs (animals 1 and 3–5), the first peak of temperature rise was observed on 4–6 dpi, followed by a decrease to normal levels over the next few days. The maximum body temperature values in pigs 1–5 were recorded from 10 to 13 dpi, ranging from 41.1 to 41.3 °C. In two out of three surviving pigs at 28 dpi, fever persisted until 26–27 dpi (Figure 1A).

The intramuscular inoculation of ASFV-PSA-1NH at a dose of 10^3^ TCID_50_ to pigs in group 2 (animals 6–10) caused fever in all animals from 5 to 21 dpi, lasting from 2 to 11 days. The maximum body temperature values in pigs that survived until 28 dpi (animals 6–9) were recorded from 9 to 13 dpi, ranging from 40.1 °C to 41.0 °C (Figure 1B).

**Figure 2:**
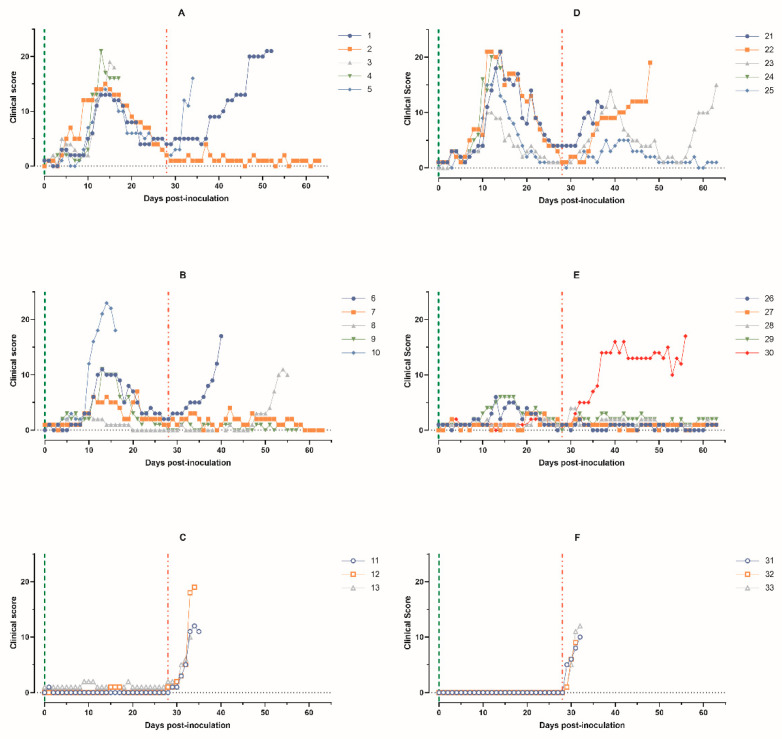
Clinical score of pigs: (**A**) animals 1–5, group 1 (intramuscularly inoculated on day 0 with ASFV-PSA-1NH at a dose of 10^5^ TCID_50_); (**B**) animals 6–10, group 2 (intramuscularly inoculated on day 0 with ASFV-PSA-1NH at a dose of 10^3^ TCID_50_); (**C**) animals 11–13, group 3 (control group intramuscularly inoculated on day 0 with PBS); (**D**) animals 21–25, group 4 (intranasally administered on day 0 with ASFV-PSA-1NH at a dose of 10^5^ TCID_50_); (**E**) animals 26–30, group 5 (intranasally administered on day 0 with ASFV-PSA-1NH at a dose of 10^3^ TCID_50_); and (**F**) animals 31–33, group 6 (control group intranasally administered on day 0 with PBS). All pigs were challenged at 28 dpi with ASFV-Stavropol 01/08. Each curve represents an individual animal’s values. Vertical dashed lines: green—days of inoculation/administration of ASFV-PSA-1NH;red—challenge with ASFV-Stavropol 01/08.

The pigs in group 1 had CS scores ≥ 6 from 6 to 24 dpi, lasting from 6 to 17 days. The maximum individual CS scores of 13 to 15 were recorded on 13–14 dpi. At 28 dpi, CS scores decreased to two to four points in the three surviving animals (Figure 2A).

Four out of five pigs in group 2 had CS scores ≥6 from 10 to 21 dpi, lasting from 3 to 10 days. The maximum individual CS scores of 7 to 23 were recorded from 13 to 21 dpi. From 22 to 28 dpi, the maximum CS scores in the four surviving pigs were zero to four points (Figure 2B).

**Figure 3:**
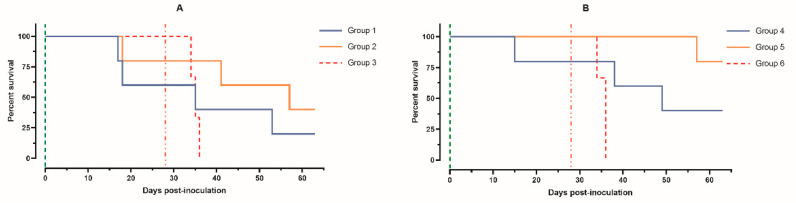
Survival rates of pigs: (**A**) group 1 (intramuscularly inoculated on day 0 with ASFV-PSA-1NH at a dose of 10^5^ TCID_50_), group 2 (intramuscularly inoculated on day 0 with ASFV-PSA-1NH at a dose of 10^3^ TCID_50_), and group 3 (control group intramuscularly inoculated on day 0 with PBS); (**B**) group 4 (intranasally administered on day 0 with ASFV-PSA-1NH at a dose of 10^5^ TCID_50_), group 5 (intranasally administered on day 0 with ASFV-PSA-1NH at a dose of 10^3.^ TCID_50_), and group 6 (control group intranasally administered on day 0 with PBS). All pigs were challenged at 28 dpi with ASFV-Stavropol 01/08. Vertical dashed lines: green—days of inoculation/administration of ASFV-PSA-1NH; red—challenge with ASFV-Stavropol 01/08.

At 28 dpi, 60% (three out of five) of the pigs in group 1 survived, while in group 2 it was 80% (four out of five) (Figure 3). All deceased animals showed an acute form of ASF. Autopsy revealed serosanguineous lymphadenitis, congestive hyperemia of the liver and kidneys with pinpoint hemorrhages, splenic hyperplasia, and lung edema.

**Figure 4:**
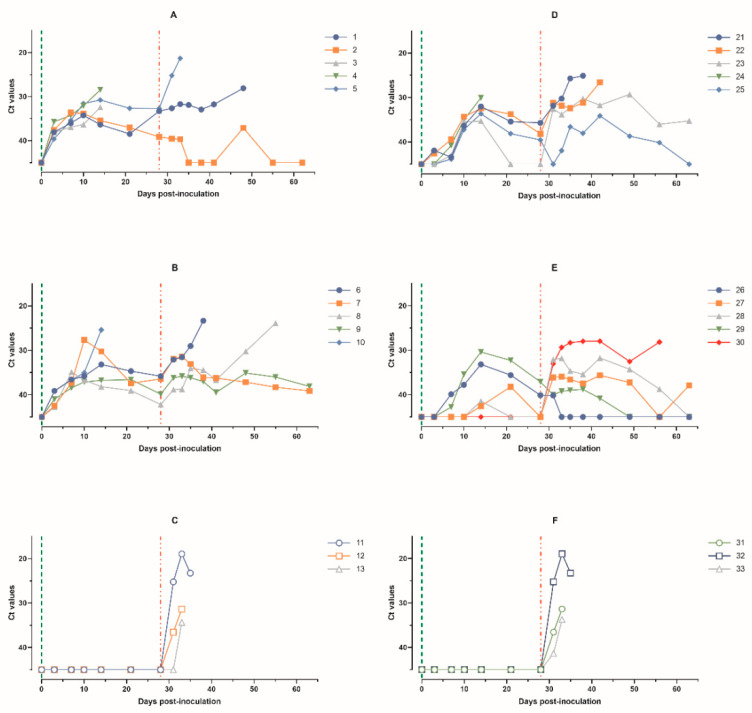
Values of ASFV-specific real-time PCR results of blood samples of pigs: (**A**) animals 1–5, group 1 (intramuscularly inoculated on day 0 with ASFV-PSA-1NH at a dose of 10^5^ TCID_50_); (**B**) animals 6–10, group 2 (intramuscularly inoculated on day 0 with ASFV-PSA-1NH at a dose of 10^3^ TCID_50_); (**C**) animals 11–13, group 3 (control group intramuscularly inoculated on day 0 with PBS); (**D**) animals 21–25, group 4 (intranasally administered on day 0 with ASFV-PSA-1NH at a dose of 10^5^ TCID_50_); (**E**) animals 26–30, group 5 (intranasally administered on day 0 with ASFV-PSA-1NH at a dose of 10^3^ TCID_50_); (**F**) animals 31–33, group 6 (control group intranasally administered on day 0 with PBS). All pigs were challenged at 28 dpi with ASFV-Stavropol 01/08. Each curve represents an individual animal’s values. Vertical dashed lines: green—days of inoculation/administration of ASFV-PSA-1NH; red—challenge with ASFV-Stavropol 01/08.

According to real-time PCR data, the pigs in group 1 had Ct values < 40 from 3 to 28 dpi, lasting from 8 to 26 days. The minimum individual Ct values were registered during 10–14 dpi. At 28 dpi, all three surviving pigs had Ct values < 40 (Figure 4A).

The pigs in group 2 had Ct values < 40 from 7 to 28 dpi, lasting from 8 to 22 days. The minimum individual Ct values were registered from 7 to 21 dpi. At 28 dpi, two out of four surviving pigs had Ct values < 40 (Figure 4B).

**Figure 5:**
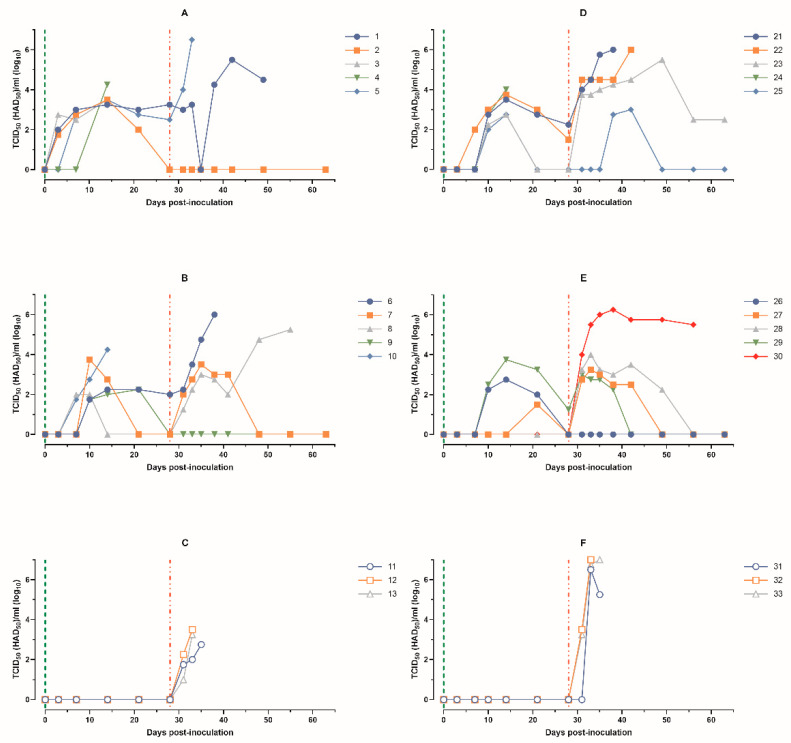
Virus titers in blood samples obtained from pigs: (**A**) animals 1–5, group 1 (intramuscularly inoculated on day 0 with ASFV-PSA-1NH at a dose of 10^5^ TCID_50_); (**B**) animals 6–10, group 2 (intramuscularly inoculated on day 0 with ASFV-PSA-1NH at a dose of 10^3^ TCID_50_); (**C**) animals 11–13, group 3 (control group intramuscularly inoculated on day 0 with PBS); (**D**) animals 21–25, group 4 (intranasally administered on day 0 with ASFV-PSA-1NH at a dose of 10^5^ TCID_50_); (**E**) animals 26–30, group 5 (intranasally administered on day 0 with ASFV-PSA-1NH at a dose of 10^3^ TCID_50_); and (**F**) animals 31–33, group 6 (control group intranasally administered on day 0 with PBS). All pigs were challenged at 28 dpi with ASFV-Stavropol 01/08. Each curve represents an individual animal’s values. Vertical dashed lines: green—days of inoculation/administration of ASFV-PSA-1NH; red—challenge with ASFV-Stavropol 01/08.

Based on the titration of blood samples in PBLC, pigs in group 1 had viremia from 3 to 28 dpi, lasting from 8 to 26 days. The titers of ASFV-PSA-1NH reached a maximum of 10^3.25^–10^4.25^ TCID_50_/mL on 14 dpi. At 28 dpi, two out of three surviving animals had viremia of 10^2.50^ and 10^3.25^ TCID_50_/mL (Figure 5A).

The pigs in group 2 had viremia from 7 to 28 dpi, lasting from 4 to 19 days. The peak viremia values of 10^2.25^–10^4.25^ TCID_50_/mL were recorded from 10 to 21 dpi (Figure 5B).

**Figure 6:**
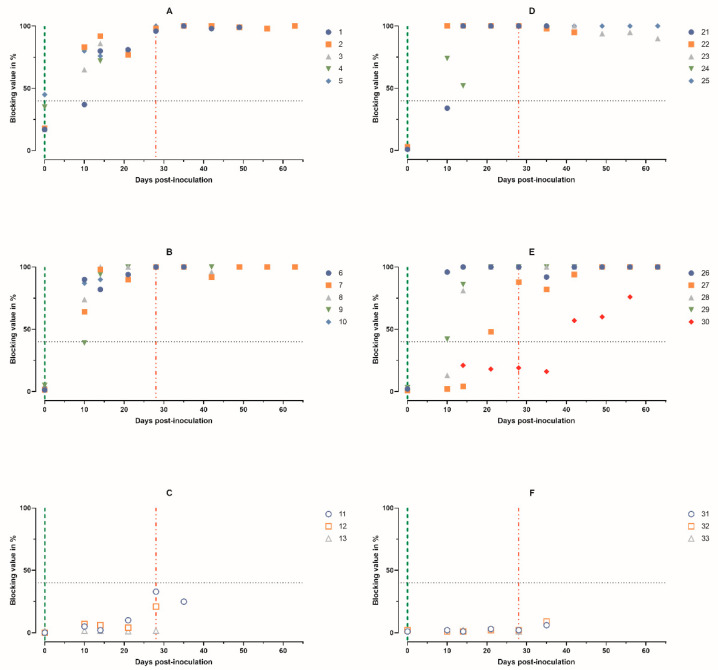
Individual kinetics of antibodies to ASFV in blood serum samples obtained from pigs: (**A**) animals 1–5, group 1 (intramuscularly inoculated on day 0 with ASFV-PSA-1NH at a dose of 10^5^ TCID_50_); (**B**) animals 6–10, group 2 (intramuscularly inoculated on day 0 with ASFV-PSA-1NH at a dose of 10^3^ TCID_50_); (**C**) animals 11–13, group 3 (control group intramuscularly inoculated on day 0 with PBS); (**D**) animals 21–25, group 4 (intranasally administered on day 0 with ASFV-PSA-1NH at a dose of 10^5^ TCID_50_); (**E**) animals 26–30, group 5 (intranasally administered on day 0 with ASFV-PSA-1NH at a dose of 10^3^ TCID_50_); and (**F**) animals 31–33, group 6 (control group intranasally administered on day 0 with PBS). All pigs were challenged at 28 dpi with ASFV-Stavropol 01/08. Each curve represents an individual animal’s values. Vertical dashed lines: green—days of inoculation/administration of ASFV-PSA-1NH; red—challenge with ASFV-Stavropol 01/08.

According to ELISA, pigs 2–5 in group 1 had diagnostically positive serum samples starting from 10 dpi, and all pigs (animals 1–5) had positive samples starting from 14 dpi. The virus-specific antibody titer in animal 4 decreased from 82% to 72% before death (Figure 6A).

In the pigs from group 2, diagnostically positive serum samples were detected from 10 to 14 dpi (Figure 6B).

After the challenge with ASFV-Stavropol 01/08, clinical signs characteristic of acute and subacute forms of ASF were observed in deceased pigs 1 and 5 from group 1, 6 and 8 from group 2, and 11–13 from group 3. Surviving pigs 2 from group 1 and 7 and 9 from group 2 had a maximum body temperature that did not exceed 40.3 °C and was recorded for no more than 2 days (Figure 1A–C).

The maximum CS values in pigs 1 and 5 from group 1 reached 21 and 16 points; in pigs 6 and 8 from group 2, 17 and 11 points; and in pigs 11–13, 10–19 points. The surviving pigs had low CS values ranging from zero to four points (Figure 2A–C). At 63 dpi, 20% (one out of five) of the pigs in group 1 survived, 40% (two out of five) in group 2, and 0% (zero out of three) in control group 3 (Figure 3A).

The minimum Ct values and maximum virus titers in blood samples from pigs 1 and 5 from group 1, 6 and 8 from group 2, and 11–13 from group 3 were recorded before their deaths. Pig 2 from group 1 did not show virus-specific DNA in the blood and showed no viremia from 55 to 63 dpi. Pigs 7 and 9 from group 2 had Ct values close to 40 units and no viremia at 55 and 63 dpi (Figure 4A–C and Figure 5A–C).

From 28 dpi until death or the end of the experiment, antibody levels were at their maximum. No diagnostically significant antibody levels were detected in animals 11–13 (Figure 6A–C).

In the organ and tissue samples of the infected pigs from group 2 (animals 6–9), the Ct values and infectivity were determined (Table 1). Positive Ct values and virus titers were observed in the samples from the deceased pigs 6 and 8. In samples from the surviving pigs 7 and 9, no infectious virus was detected.

### 3.2. Immunobiological Indicators in Pigs after Intranasal Administration of Different Doses of ASFV-PSA-1NH and Intramuscular Challenge with ASFV-Stavropol 01/08

The intranasal administration of pigs 21–25 from group 4 with ASFV-PSA-1NH at a dose of 10^5^ TCID_50_ caused fever in all animals from 3 to 23 dpi, lasting from 10 to 18 days, with maximum individual temperature values ranging from 40.9 to 42.0 °C (Figure 1D).

The intranasal administration of pigs 26–30 from group 5 with ASFV-PSA-1NH at a dose of 10^3^ TCID_50_ caused fever in four out of five animals from 10 to 24 dpi. Pigs 26 and 29 had fever for 11 and 15 days, respectively, with maximum individual temperatures of 41.2 and 41.4 °C. Pigs 27 and 28 had fever for 3 days and 1 day, respectively, with maximum individual temperatures of 40.3 and 40.0 °C. Pig 30 had a body temperature < 40.0 °C from 0 to 28 dpi (Figure 1E).

The pigs in group 4 had individual CS scores ≥6 from 8 to 24 dpi, lasting from 6 to 16 days. The maximum individual CS scores, ranging from 10 to 21 points, were recorded from 11 to 14 dpi. From 25 to 28 dpi, surviving pigs in group 4 had CS scores of one to five points (Figure 2D).

The pigs in group 5 had individual CS scores ≥ 6 in animals 26 and 29 from 13 to 17 dpi, lasting from 1 to 4 days. CS scores ≥ 6 were not established in pigs 27, 28, and 30 from 0 to 28 dpi (Figure 2E).

During the period from 0 to 28 dpi, the survival rate of the pigs after the administration of ASFV-PSA-1NH was 80% (four out of five) in group 4 and 100% (five out of five) in group 5 (Figure 3). Pig 24 from group 4 developed an acute form of ASF.

In blood samples from pigs in group 4, Ct values < 40 in real-time PCR were registered from 7 to 28 dpi, lasting from 5 to 22 days. The minimum individual Ct values were recorded on 14 dpi (Figure 4D).

In blood samples from pigs 26 and 29 in group 5, Ct values < 40 were observed from 10 to 28 dpi, lasting from 15 to 19 days, respectively, with minimum Ct values on 14 dpi. Pig 27 had Ct < 40 only on 21 dpi. Pigs 28 and 30 had Ct values > 40 from 0 to 28 dpi (Figure 4E).

The kinetics of viremia corresponded to the results of real-time PCR. The pigs in group 4 had viremia from 7 to 28 dpi, lasting from 5 to 19 days. The titers of ASFV-PSA-1NH reached a maximum of 10^2.75^–10^4.00^ TCID_50_/mL on 14 dpi. At 28 dpi, viremia was only detected in pigs 21 and 22, with titers of 10^2.25^ and 10^1.50^ TCID_50_/mL, respectively (Figure 5D).

In group 5, relatively long viremia, from 12 to 19 days, was observed in animals 26 and 29 from 10 to 28 dpi, with a peak on 14 dpi, with titers of 10^2.75^ and 10^3.75^ TCID_50_/mL, respectively. Pig 27 had viremia only on 21 dpi, with a titer of 10^1.50^ TCID_50_/mL. Viremia was not detected in animals 28 and 30 (Figure 5E).

Serum samples from pigs in group 4 were diagnostically positive from 10 to 14 dpi. Before death, the level of virus-specific antibodies in pig 24 decreased from 74% to 52% (Figure 6D). Serum samples from pigs 26, 28, and 29 in group 5 became diagnostically positive from 10 to 14 dpi, and pig 27 became positive from 28 dpi. Pig 30 did not have diagnostically positive samples throughout the period of 0–28 dpi (Figure 6E).

After challenge with ASFV-Stavropol 01/08, all the pigs 21–25 from group 4 and 31–33 from control group 6 showed fever. The body temperature of pigs 26–28 from group 5 remained normal until 63 dpi, and pig 29 had three short-term temperature elevations ranging from 40.1 to 40.3 °C. Pig 30 had fever from 31 dpi until death at 57 dpi (Figure 1D–F).

Pigs 21 and 22 from group 4 and 31–33 from group 6 showed an acute form of ASF. Pig 23 had a chronic form of the disease. Only one pig from group 4, pig 25, had low CS values. Pigs 26–29 from group 5 had CS values that did not exceed two to three points. Pig 30 showed a subacute form of ASF from 35 to 57 dpi (Figure 2D–F). In the end, 40% (two out of five) of the pigs in group 4 survived, 80% (four out of five) in group 5, and 0% (zero out of three) in group 6 (Figure 3B).

In blood samples from pigs 21 and 22 in group 4, the minimum Ct values were registered before their deaths. The surviving pigs, 23 and 25, had Ct values < 40 from 31 to 63 and 35 to 49 dpi, respectively (Figure 4D). In blood samples from pigs 26 and 29 in group 5, the Ct values were around or greater than 40 until 63 dpi. Pigs 27 and 28 had Ct values < 40 from 31 to 49 and 56 dpi, respectively. On 63 dpi, no virus-specific DNA was detected in the blood samples from pigs 26–29 (Figure 4E). Pig 30 had Ct values < 40 from 31 until the day of death at 57 dpi.

In blood samples from pigs 21 and 22 in group 4, viremia was registered from 31 dpi, reaching levels of 10^5.50^–10^6.00^ HAD_50_/mL before their deaths. Pig 23 had viremia from 31 to 63 dpi. Pig 25 had a peak viremia of 10^3.00^ HAD_50_/mL at 42 dpi, and no infectious virus was detected from 49 to 63 dpi (Figure 5D).

Pig 26 from group 5 did not show detectable infectious virus until 63 dpi. Animals 27–29 had viremia from 31–38 to 49 dpi, with titers ranging from 10^2.25^ to 10^4.00^ HAD_50_/mL. No infectious virus was detected in blood samples taken from these pigs at 56 and 63 dpi. Pig 30 had viremia from 31 dpi until the day of death (Figure 5E).

Challenge with ASFV-Stavropol 01/08 did not affect the levels of virus-specific antibodies in the pigs from groups 4 and 5 (except 30). Note that in the serum of pig 30 from group 5, diagnostically positive virus-specific antibodies were recorded starting at 42 dpi. Diagnostically positive serum samples were not detected in animals 31–33 (Figure 6D–F).

The results of organ samples from the surviving pigs 26–29 from group 5 are presented in Table 2. The presence of infectious virus was confirmed in the tonsils of animals 27 and 28 and in the salivary glands of pig 28.

## 4. Discussion

The choice for these studies with ASFV-PSA-1NH and ASFV-Stavropol 01/08 was based on the following considerations. ASFV-PSA-1NH and ASFV-Stavropol 01/08 are heterologous: the former belongs to immunotype IV and genotype I, while the latter belongs to seroimmunotype VIII and genotype II [17]. Both genotype I and genotype II ASFVs were found in pigs in China in 2021 [33]. The origin of the two detected non-hemadsorbing genotype I ASFVs, HeN/ZZZ-P1/21 and SD/DY-I/21, would become clearer after obtaining results about their immunotype in an in vivo immunologic test [16]. Some non-hemadsorbing natural isolates and laboratory recombinants have shown the ability to provide heterologous protection [23,24]. The ASFV-PSA-1NH-inoculated pigs after challenge with ASFV-Stavropol 01/08 can be differentiated based on hemadsorption.

In 19 out of 20 pigs (groups 1, 2, 4, and 5), the administration of ASFV-PSA-1NH was confirmed by immunobiological indicators. For one pig (group 5, animal 30), infection with a dose of 10^3^ TCID_50_ intranasally was not confirmed. This was indicated by the absence of fever, viral DNA, and infectious virus in the blood samples, as well as the absence of diagnostically significant levels of antiviral antibodies in the serum. Therefore, we considered the number of animals included in group 5 to be four (animals 26–29). The main argument for this was the seroconversion test. The absence of diagnostically positive levels of virus-specific antibodies in the serum from 0 to 28 dpi for ASFV-PSA-1NH indicated unsuccessful inoculation. Similar cases have been described in other studies [34]. However, it can be hypothesized that animal 30 may have been exposed to ASFV-PSA-1NH in a negligible dose, as indicated by the level of blocking in the ELISA from day 14 to 28 dpi, which ranged from 20 to 25%, while the control animals 31–33 showed levels near 0%. This hypothesis is supported by the duration of illness in animal 30, which lasted for 29 days after challenge with ASFV-Stavropol 01/08.

From our results, it can be concluded that at a dose of 10^5^ TCID_50_, the route of administration, either intranasal or intramuscular, did not have a significant impact on the investigated immunobiological characteristics in the pigs. The parameters of rectal temperature, clinical signs, presence of viral DNA and viremia in blood samples, and survival rates were similar or close (Table 3). The infection progressed in a subacute or chronic form, and the percentage of protection against heterologous ASFV-Stavropol 01/08 ranged from 20% to 40%. Similar percentages of protection against mortality were observed in the pigs inoculated with the attenuated hemadsorbing strain ASFV-Katanga-350 (seroimmunotype I, genotype I) and subsequently challenged with ASFV-Stavropol 01/08 [35].

In the intranasal route of administration at a dose of 10^3^ TCID_50_ of ASFV-PSA-1NH, it met the minimum safety requirements of the World Organisation for Animal Health (WOAH): no pig reached the humane endpoint (euthanasia) or died from ASF-related causes; all the pigs in the group had an average body temperature increase of 0.5 °C compared with the baseline level (*p* < 0.05); no individual pig showed a temperature increase exceeding 2.5 °C above the baseline level for a period longer than 3 days; and compared with groups 1, 2, and 4, the clinical signs in group 5 were significantly lower (*p* < 0.05). The maximum viremia did not exceed 10^3.75^ TCID_50_/mL (Table 3). The intranasal administration of ASFV-PSA-1NH at a dose of 10^3^ TCID_50_ provided 100% protection (of those who responded positively to the vaccination according to real-time PCR and ELISA results) against death after intramuscular challenge with heterologous ASFV-Stavropol 01/08 at a dose of 10^3^ HAD_50_ on day 28. It should be noted that this study did not investigate transmission issues.

Usually, the incubation period tends to be shorter after intradermal and intramuscular inoculation compared with oronasal or oral infections. Higher doses of the virus mostly result in shorter incubation periods compared with lower doses [36,37,38]. This was also observed in our experiments. The results presented in Table 3 indicate that when ASFV-PSA-1NH was administered intranasally at a dose of 10^3^ TCID_50_, the onset of clinical signs and viremia occurred 3–5 days later compared with intramuscular inoculation. Overall, our results were consistent with previous studies using non-hemadsorbing isolates OURT88/3 and NH/P68 [26,27]. The combination of a low dose of ASFV-PSA-1NH and intranasal administration contributed to the development of heterologous immune protection.

The comparison of data on body temperature kinetics and clinical signs of disease in the pigs from groups 1, 2, 4, and 5 (Table 3) indicated a longer incubation period following the intranasal administration of ASFV-PSA-1NH at a dose of 10^3^ TCID_50_. Differences in vaccine virus replication and protection depending on the inoculation route and dose can be attributed, in our opinion, to several reasons. First, intranasal virus administration reduces the number of initially infected macrophages through non-specific defense mechanisms and as a result of replication of the attenuated virus in the local mucosal entry site stimulates the induction of a systemic immune response. Second, the binding of ASFV particles to red blood cells facilitates virus dissemination in the infected pigs [39,40,41]. In the pigs infected with hemadsorbing isolates of ASFV, 90% of the virus in the blood is associated with the red blood cell fraction. In contrast, in the pigs infected with non-hemadsorbing isolates, only a small percentage of the virus is associated with red blood cells [39]. As a result, virus dissemination in the pig’s body is reduced, creating favorable conditions for the development of immune protection. Third, protection against ASF is mediated by the sequential involvement of effector cells of the cellular immune response. This includes natural killer (NK) cells, antibody-dependent cell-mediated cytotoxicity (ADCC), and cytotoxic T lymphocytes (CTLs) [27,42,43,44,45,46]. It has been reported that ADCC plays a more significant role in suppressing ASF virus replication than CTL [46].

A significant factor in the formation and implementation of protective immune responses is the serotype-specific hemadsorbing glycoprotein CD2v (gp 110–140), which is located on the plasma membrane of infected macrophages and, consequently, on the outer envelope of the virion [47,48]. This glycoprotein, apparently, contains epitopes that determine the formation of both homologous and heterologous antigenic determinants (epitopes) [47]. Hypothetically, during the replication of hemadsorbing isolates/strains of ASFV, the presence of a significant “carbohydrate cloud” around CD2v limits the number of accessible epitopes for immune cells. Furthermore, the interaction between pig erythrocyte receptors and CD2v oligosaccharides leads to hemadsorption [49]. As a result, erythrocytes create a steric effect that hinders the interaction of CD4^+^ and CD8^+^ cells with antigen-presenting macrophages. Deletions or frame shifts in CD2v can result in the loss of glycosylation sites and the ability to induce hemadsorption. This may increase the proportion of heterologous T and B cell epitopes on CD2v and enhance the interaction of CD4^+^ and CD8^+^ cells with antigen-presenting macrophages. Ultimately, this expands the range of immune cells capable of forming and implementing effective defense mechanisms. Multiple linear B cell epitopes of CD2v have recently been identified [50].

Under natural conditions, ASFV is most transmitted among pigs via contact with excreted viral particles through nuzzling or ingestion [51]. Experimental oronasal inoculation mimics natural infection because it results in uptake of the ASFV via the oral and upper respiratory mucosa; the virus interacts with mucosal surfaces and is exposed to innate defense mechanisms [38]. Therefore, despite the challenges in standardizing the dosage for intranasal administration, this route appears to be a viable alternative for ASF vaccination.

Recently, WOAH published the Draft Standards for ASF Modified Live Virus (MLV) Vaccines for Domestic and Wild Pigs on its website (https://www.woah.org/en/document/report-of-the-meeting-of-the-woah-biological-standards-commission-september-2023/, accessed on 21 April 2024), designating MLV vaccines as the first generation of ASF vaccines. Some of our results and arguments could be useful in their discussion.

The presence of ASFV-Stavropol 01/08 in the tonsils and salivary glands at 63-dpi (Table 2) indicates the need to extend the observation period for animals. In these studies, out of 20 pigs inoculated with ASFV-PSA-1NH, 16 pigs were challenged with ASFV-Stavropol 01/08 at 28 dpi. Among them, seven pigs died from ASF, including three pigs between 49–63 dpi. Out of the nine surviving pigs, one pig developed chronic ASF and became sick again on day 57, with a body temperature of 41.6 °C at the end of the experiment. Thus, out of the eight deceased and fatally diseased pigs, four (50%) experienced this outcome between 49–63 dpi. These results confirm the justification of WOAH’s proposal to establish a minimum observation period of at least 60 days after vaccination. Furthermore, it has been reported that in most pigs that survived after intramuscular or intranasal infection with moderately virulent isolates Malta’78 and Netherlands’86, blood and oral swab samples remained positive in real-time PCR for at least 70 days [34]. During the time period from 64 to 93 dpi, the percentages of positive samples for nucleic acid detection of an attenuated ASFV strain MK-200 in blood, oral fluids, buccal swabs, and tonsillar scrapings of the pigs reached 27%, 41%, 23%, and 36%, respectively [52]. The NH/P68-inoculated pigs had the virus isolated from the tissues up to 99 dpi [42].

Various methodologies are used to assess clinical signs of ASF [12,35,38,42]. We preferred the methodology of King et al., 2011 [12], which should ideally be supplemented with neurological signs (limb paresis and paralysis). This methodology allows for the comparison of clinical signs in the animals infected with ASFV isolates/strains of different virulence.

We find it debatable to state, as WOAH does, that “currently acceptable efficacy should be demonstrated against the pandemic genotype II, lineage *B646L* (p72) virus, which is currently circulating widely among domestic pigs and wild boars”. The genotype II classification does not determine immunological compatibility. We propose selecting homologous/heterologous isolates/strains for studying the efficacy of candidate vaccines based on the seroimmunotypic classification of ASFV, specifically the pandemic virus belonging to seroimmunotype VIII. Alternatively, both classifications of ASFV can be considered. This proposal is based on the fact that among ASFV isolates belonging to genotype I, isolates of seroimmunotypes I and IV were present on the Iberian Peninsula [7,35]. Among the ASFV VIII seroimmunotype isolates, there are isolates Georgia07 and Stavropol 01/08 belonging to genotype II and Rhodesia isolate belonging to genotype VIII [7].

Real-time PCR is effective for the preliminary determination of viremia, virus shedding, and the presence of the virus in organ samples. The final result should be determined based on in vitro or in vivo infectivity.

## 5. Conclusions

This study characterized the immunobiological properties of the ASFV-PSA-1NH isolate.

We noted that at high doses (10^5^ TCID_50_), the route of administration, either intranasal or intramuscular, did not have a significant impact on the investigated immunobiological characteristics: the disease typically progressed in subacute or chronic forms, and the percentage of protection against heterologous ASFV-Stavropol 01/08 was 20–40%.

Intranasal administration at a dose of 10^3^ TCID_50_ of ASFV-PSA-1NH met the minimum safety requirements and provided 100% protection (of those who responded positively to the vaccination according to real-time PCR and ELISA results) against death after intramuscular challenge with heterologous ASFV-Stavropol 01/08 at a dose of 10^3.^ HAD_50_ on day 28. However, the obtained results do not warrant classifying ASFV-PSA-1NH as a candidate vaccine strain. They only emphasize the need for comprehensive research on the properties of candidate vaccines.

As recommendations for testing first-generation candidate vaccines, we propose (i) selecting homologous/heterologous strains for studying the efficacy of candidate vaccines based on the seroimmunotypic classification of ASFV, (ii) extending the observation period for pigs to 90 dpi, and (iii) evaluating vaccination based on seroconversion test results.

## Figures and Tables

**Table 1:** Detection of ASFV-Stavropol 01/08 genome (Ct) and virus titers (lg HAD_50_/0,1 g) in organ samples of the pigs from group 2: dead (animals 6 and 8) and survivors (animals 7 and 9) after challenge with ASFV-Stavropol 01/08.

Organs	Number of Pigs							
	6		7		8		9	
	Ct	Titers	Ct	Titers	Ct	Titers	Ct	Titers
Spleen	29.02	5.25	N/A *	0.00	18.84	6.50	38.70	0.00
Lungs	20.77	4.50	N/A	0.00	18.38	4.75	32.47	0.00
Tonsils	26.20	3.25	N/A	0.00	22.60	2.50	N/A	0.00
Salivary glands	28.23	3.25	41.64	0.00	20.01	6.00	40.22	0.00
L/n ** mesenteric	22.60	4.25	N/A	0.00	24.11	2.75	N/A	0.00
L/n portal	23.05	4.75	N/A	0.00	22.10	3.50	N/A	0.00
L/n parotid	27.45	4.75	44.42	0.00	27.45	4.25	36.79	0.00
L/n submandibular	23.14	4.00	N/A	0.00	41.47	2.50	N/A	0.00

*—not available, **—Lymph node.

**Table 2:** Detection of ASFV-Stavropol 01/08 genome (Ct) and virus titers (lg HAD_50_/0,1 g) in organ samples of pigs 26–29 from group 5 that survived after challenge with ASFV-Stavropol 01/08.

Organs	Number of Pigs							
	26		27		28		29	
	Ct	Titers	Ct	Titers	Ct	Titers	Ct	Titers
Spleen	N/A *	0	36.29	0	36.52	0	N/A	0
Lungs	N/A	0	36.13	0	40.43	0	N/A	0
Tonsils	41,27	0	35.95	1.75	34.30	1.75	38.04	0
Salivary glands	43.88	0	39.03	0	36.01	1.75	38.56	0
L/n ** mesenteric	N/A	0	39.96	0	42.91	0	N/A	0
L/n portal	40.20	0	36.54	0	39.45	0	42.58	0
L/n parotid	39.54	0	43.59	0	44.22	0	39.30	0
L/n submandibular	38.92	0	40.35	0	37.44	0	42.11	0

*—not available, **—Lymph node.

**Table 3:** The influence of the dose and route of administration of ASFV-PSA-1NH virus on the immunobiological indicators in the pigs during 0–28 days.

Indicators	Intranasal, TCID_50_		Intramuscular, TCID_50_	
	10^5^ Group 4	10^3^ Group 5	10^5^ Group 1	10^3^ Group 2
Rectal temperature				
Baseline temperature, °C	39.0 ± 0.3	39.0 ± 0.3	39.1 ± 0.2	39.2 ± 0.2
Fever (≥40 °C)/total pigs	5/5	4/4	5/5	5/5
Period, dpi	3–23	10–24	4–27	5–21
Duration, days	10–18	1–15	6–18	2–11
Mean values, °C (0–28 dpi)	40.1 ± 0.8	39.5 ± 0.6	39.9 ± 0.7	39.6 ± 0.7
Maximum values, °C (0–28 dpi)	40.9–42.0	40.0–41.4	41.1–41.6	40.0–41.8
Survival rate after administration of ASFV-PSA-1NH				
Survived/total pigs	4/5	4/4	3/5	4/5
Clinical signs (scores)				
Manifestation (≥6 scores)/total	5/5	2/4	5/5	4/5
Period, dpi	7–25	13–17	6–24	10–21
Duration, days	6–18	0–5	6–17	0–10
Scores, M ± m	6.3 ± 6.1	1.6 ± 1.4	6.4 ± 5.3	3.6 ± 4.5
Maximum scores	10–21	3–6	13–15	3–11
Real-Time PCR				
Manifestation (Ct < 40)/total)	5/5	3/4	5/5	5/5
Period, dpi	7–28	7–28	3–28	7–28
Duration, days	5–22	0–19	26	22
Ct values, M ± m	39.6 ± 4.9	>40.0	36.6 ± 4.5	37.9 ± 4.8
Minimum Ct values	30.1–35.3	30.4–>40.0	26.9–36.3	25.4–36.6
Viremia				
Manifestation (>2.0 TCID_50_/mL)/total	5/5	3/4	5/5	5/5
Period, dpi	10–28	10–21	3–28	7–28
Duration, days	5–19	0–12	18–26	4–15
TCID_50_/mL, M ± m	10^1.24^ ± 10^1.45^	10^0.55^ ± 10^1.09^	10^1.88^ ± 10^1.49^	10^1.02^ ± 10^1.29^
Maximum values, TCID_50_/mL	10^2.75^–10^4.00^	10^2.75^–10^3.75^	10^3.00^–10^3.75^	10^2.25^–10^4.25^

## Data Availability

The original contributions presented in the study are included in the article and Supplementary Material, further inquiries can be directed to the corresponding authors.

## Supplementary Materials

The following supporting information can be downloaded at https://www.mdpi.com/article/10.3390/ani14091277/s1, Figure S1. Scheme of experiment 1 on intramuscular inoculation of material (0 days post-infection, dpi) until the end of the experiment (63 dpi). Figure S2. Scheme of experiment 2 on intranasal administration of material (0 dpi) until the end of the experiment (63 dpi).
