# Supplementary figures and images for: Administration Routes and Doses of the Attenuated African Swine Fever Virus Strain PSA-1NH Influence Cross-Protection of Pigs against Heterologous Challenge
Source: Animals (Basel). 2024 Apr 24;14(9):1277. doi: 10.3390/ani14091277 (PMC11083577; doi:10.3390/ani14091277)

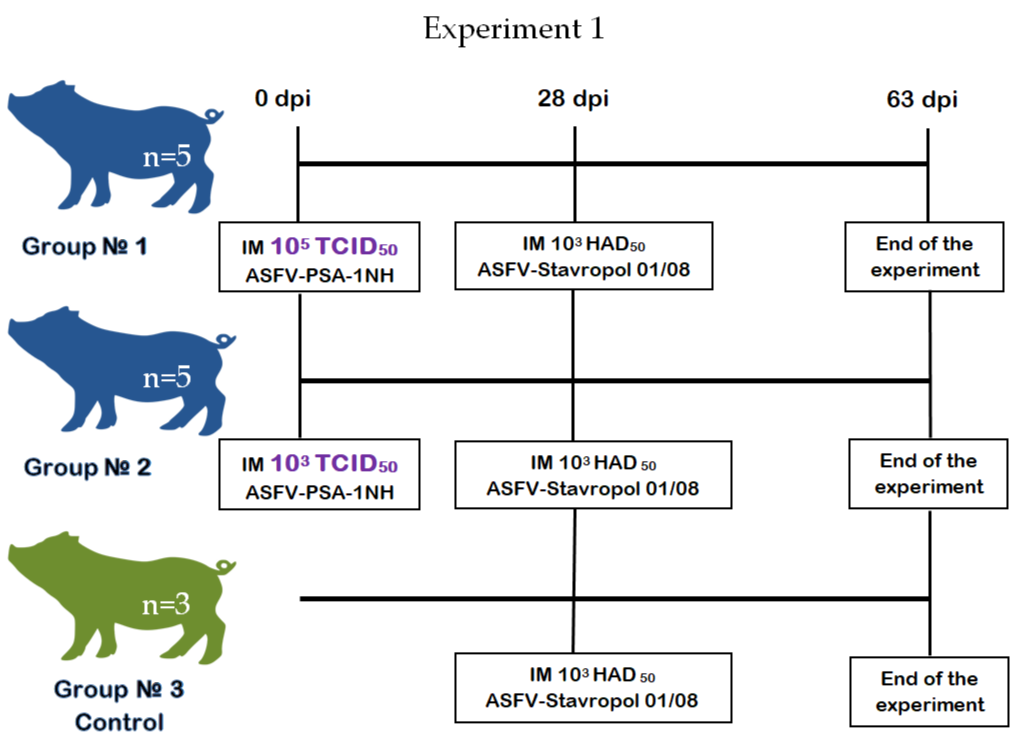

Supplement: Supplementary file 1 [file animals-14-01277-s001.zip › Figure S1. Scheme of the experiment 1 on intramuscular inoculation of marerial (0 days post-infection, dpi) until the end of the experiment (63 days post-infection, dpi)..png]

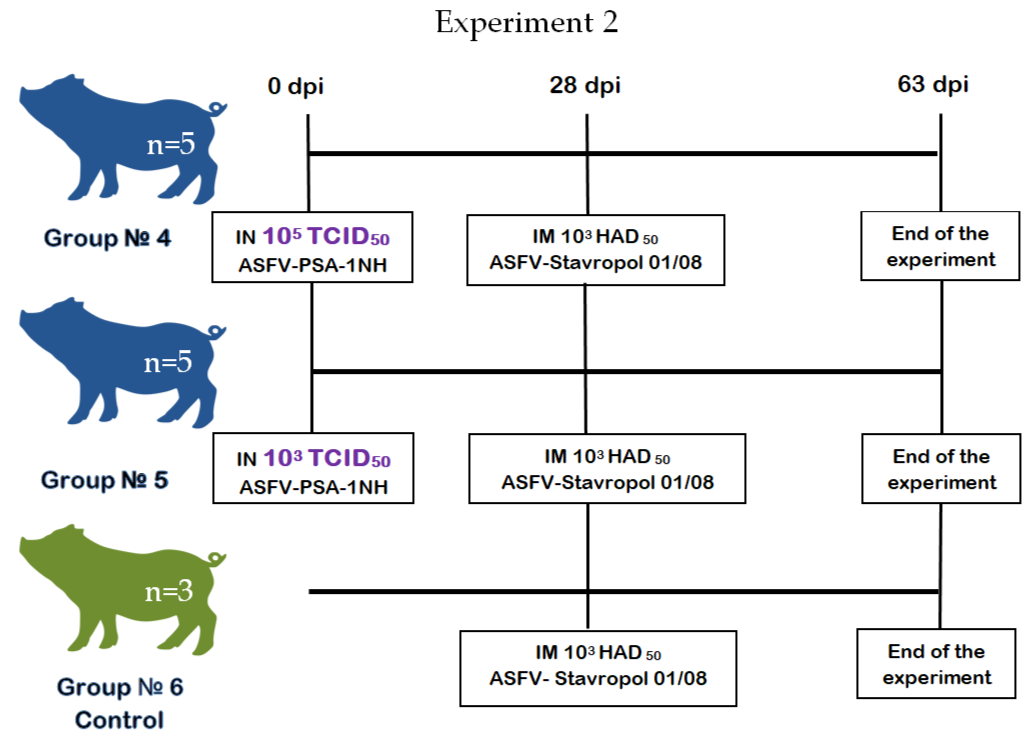

Supplement: Supplementary file 1 [file animals-14-01277-s001.zip › Figure S2. Scheme of the experiment 2 on intranasal administration of material (0 days post-infection, dpi) until the end of the experiment (63 days post-infection, dpi)..png]
